# Consumption of fresh or minimally processed foods and ultra-processed foods according to social vulnerability among Brazilian university students

**DOI:** 10.3389/fpubh.2025.1680049

**Published:** 2025-09-26

**Authors:** Maria Eduarda Ribeiro José, Natália Oliveira, Patrícia Maria Périco Perez, Nayara Côrtes Rocha, Daniela Silva Canella

**Affiliations:** ^1^Postgraduate Program in Food, Nutrition, and Health, Rio de Janeiro State University, Rio de Janeiro, Brazil; ^2^Institute of Nutrition, Rio de Janeiro State University, Rio de Janeiro, Brazil; ^3^Postgraduate Program in Human Nutrition, University of Brasília, Brasília, Brazil

**Keywords:** food consumption, food processing, food security, socioeconomic status, quotas, university

## Abstract

**Introduction:**

The COVID-19 pandemic worsened the diet quality of the population, including young adults, owing to the sudden economic insecurity caused by global chaos.

**Objective:**

To describe the food consumption of students admitted to university after the pandemic, according to the social vulnerability situation.

**Methods:**

Cross-sectional study conducted with students who were in a Brazilian public university located in Rio de Janeiro in 2022. Data collection was carried out online. Fresh or minimally processed foods and ultra-processed foods were assessed considering the consumption on the previous day and the regular intake (>5 days/week). To assess social vulnerability three indicators were used: the form of university admission, a socioeconomic score, and food (in)security.

**Results:**

A total of 924 students participated in the study. Most of them were 18–22 years-old (50%), cisgender women (62%), of white people (53%), 30% were quota students, 32% had family income ranging between 2 to 5 minimum wages, and 36% were food insecure, with or without hunger. Consumption of fresh or minimally processed foods was more frequent among students with a higher socioeconomic score, non-quota students, and food-secure ones. Consumption of ultra-processed foods seems to have been less influenced by students’ social conditions in this sample of students.

**Conclusion:**

The food consumption, mainly related to fresh or minimally processed foods, was influenced by the vulnerability of university students. The findings reinforce the need for permanent student support policies, since universities can be strategic places to ensure the human right to adequate and healthy food.

## 1 Introduction

The COVID-19 pandemic was declared a Public Health Emergency of International Concern by the World Health Organization in 2020 and lasted until 2023. It had a huge impact on health, causing sudden changes in people’s lives through physical distancing, and leading to social and economic consequences, e.g., increased social vulnerability (1), which can be characterized by cultural, social and economic aspects that determine the opportunities to access goods and services (2). Changes in routine, including COVID-19-related fear and anxiety, absence of face-to-face activities and increased online food purchases seem to have affected eating habits. Studies have reported worsened diet quality during physical distancing (3–5).

Adolescents and young adults are part of the population whose diet was affected. An increased consumption of beans, fruits and vegetables was reported, and it can be explained by the fact that people had more free time to cook, although this apparently did not increase the overall diet quality. Still, there was a higher consumption of sweets, probably owing to boredom and distancing-induced stress (6). A Brazilian study conducted in the pre-pandemic period (2017–18) found that ultra-processed foods contributed more to the daily energy intake of younger individuals, and their consumption was positively associated with schooling/education, which is one of the determinants of health (7).

Studies focusing on university students reported a high intake of ultra-processed foods, but low consumption of fresh or minimally processed foods. They often skip breakfast and replace dinner with snacks (8, 9). In Brazil, pre-pandemic studies had already highlighted unhealthy eating habits among university students, characterized by frequent consumption of fast food, snacks, sweets, sugary beverages, and low intake of fruits and vegetables (8, 10–13). Evidence about university students found that unhealthy food practices were more present among food-insecure students (14). Furthermore, the frequency of food insecurity among this population ranged from 21 to 82%, that is, food insecurity is highly present in this public and it seems to be associated with the lack of consumption of some fresh or minimally processed foods, which can be replaced by ultra-processed foods, probably leading to a lower intake of micronutrients (15, 16).

The risk of food insecurity is associated with many factors, including income, which was further impaired during the COVID-19 pandemic. Economic insecurity caused by the global chaos of the pandemic can have lasting consequences for the population regarding social, health and life-related conditions. Poverty, and consequently social vulnerability, hinders access to safe and nutritious food, resulting in a high prevalence of food insecurity. Social vulnerability represents multiple determinants that impact the fact that citizens live in fragility or lack access to rights, such as the right to adequate food, which is weakened in situations of insecurity (17, 18). In Japan, for example, almost half of working students lost their jobs during the pandemic, affecting their lives, studies and health, leaving them in a state of vulnerability (19). Exploring how the COVID-19 pandemic has impacted the diet and food security of university students, can help identify long-term health risks associated with a poor diet and increased consumption of ultra-processed foods, providing essential data for the development of public policies and interventions in the universities to promote food security and health. In this context, the objective of this study was to describe the food consumption of students admitted to university after the pandemic, according to the social vulnerability situation.

## 2 Materials and methods

Cross-sectional study, conducted with students who were admitted to a Brazilian public university located in the state of Rio de Janeiro (Rio de Janeiro State University - UERJ) in 2022. This year was defined for data collection because it coincides with the return of face-to-face activities in the university, after the COVID-19 pandemic, a period that may have influenced eating perceptions and behaviors. A total of 4,751 undergraduate students entered the 12 campuses of the university in 2022 (20) and all of them, over 18 years of age, were eligible for the study and were invited to participate.

The main strategy used for attracting students was sending an invitation by email to ask them to participate in the study. Their e-mail addresses were provided by UERJ’s Office of the Dean for Student Policies and Assistance. The departments of all undergraduate degree courses were also notified by e-mail about the study and requested to help disseminate information about it to their students and a social media profile was created to disseminate information about the study and enable interaction with students. Additionally, on the Maracanã campus, which is considered the main one at the university, the students were approached directly by the research team in classes, events, the university restaurant, and academic centers of the different courses. To provide direct access to the form, cards with a QR code were handed out, and posters with the QR code were distributed across the campus.

Data collection was carried out online, with a self-applicable form available in the Google Forms platform. The collection for first-semester students took place between June and October 2022, and for second-semester ones, from October 2022 to February 2023. The data collection form was designed with open and closed questions and divided into 10 blocks: 1- Personal data and sociodemographic characteristics, 2- Nutritional status and health, 3- Food consumption on the previous day, 4- Food practices, 5- Food and nutrition security, 6- Behaviors, 7- Non-communicable diseases, 8- Sleep, 9- Depression, Anxiety and Stress and 10- Household goods. The questionnaire was designed with questions previously used in national health surveys and validated scales. Also, the questionnaire was piloted, and adjustments were made to the sequence of questions.

The variables of interest of the present study are related to food consumption and social vulnerability. The assessment of the consumption of fresh or minimally processed foods and ultra-processed foods was based on two independent but complementary strategies: the intake on the previous day (21) and the regular intake (at least 5 from the previous 7 days - > 5 days/week) (8). Both strategies are adopted in Brazilian health surveys (22, 23) and can contribute to presenting a broader scenario of diet quality.

For consumption on the previous day, individuals were asked about 12 fresh or minimally processed items and 13 ultra-processed items. Subsequently, based on the sum of positive responses for the items of each group, two counting scores were calculated: one for consumption of fresh or minimally processed foods (it may range from 0 to 12) and one for consumption of ultra-processed foods (it may range from 0 to 13). This instrument was designed considering the foods most consumed by the Brazilian population according to data from the Household Budget Survey (24), and it was previously validated (24, 25).

For the regular consumption, individuals were asked about 4 fresh or minimally processed food markers and 8 ultra-processed food markers. Based on the weekly frequency of consumption of each marker, the frequency of regular consumption (at least five of the previous 7 days) of the foods was assessed (8).

Social vulnerability was analyzed considering three composed indicators: (1) a socioeconomic score, (2) the form of university admission, and (3) the food (in)security situation, which is a consequence of the social vulnerability but is also a marker of vulnerability.

The socioeconomic score considers household goods (radio, refrigerator, media player, washing machine, microwave, landline, computer, air conditioning, TV, internet on cell phone, cars) and mother’s level of education, and the points are used to generate a score that ranges between 0 and 26 (26), which was divided into tertiles (T). Scores were not calculated for students who did not know or did not want to answer any of the questions.

As for form of admission to university (reservation of vacancies/quotas from the entrance exam, fierce competition via the entrance exam, or other), the students were grouped into quota and non-quota students. The quota system promotes admission via entrance exams based on economic (income), racial (black and brown), and educational criteria (origin from public schools), among others (27).

To evaluate the situation of food and nutrition security, a validated scale for the Brazilian adolescents was used. The instrument is composed of seven questions, generating a score from 0 to 6, calculated by the sum of affirmative answers (yes answer). For the first four and sixth questions, an affirmative answer corresponds to one point. For the fifth item of the questionnaire, the score follows the following criteria: when it occurs “often” or “sometimes,” one point is added; when “few times” or “I do not know” occurs, there is no punctuation. After adding up the points and creating the score, individuals who reached up to one point were considered food secure; those who scored between two and four points were classified as food insecure without hunger; and when the score resulted in five or six points, individuals were classified as food insecure with hungry (28).

The following variables were used for the characterization of the sample: age (grouped in 18–22, 23–29, 30 and more), campus (Maracanã or other campuses), gender (cisgender woman, cisgender man, transgender man, non-binary person, other, rather not say), race/skin color (white, brown, black, Asian), area from the undergraduate degree course (biomedical, education and humanities, social sciences, technology and science), paid work (yes or no), mother’s level of education (none, incomplete elementary school, complete elementary school/incomplete high school, complete high school, complete or incomplete higher education, unsure), household arrangement (living alone; with relatives, not necessarily parents; with other people who are not family members), family income (up to 1 minimum wage - MW, 1–2 MW, 2–5 MW, 5–10 MW, more than 10 MW, in 2022, the Brazilian minimum wage was R$ 1,212, which in 2024 is equivalent to US$ 210).

The completed answers were exported to an Excel spreadsheet and turned into Stata databases. All statistical analyses were performed using the statistical package Stata/SE version 16.0 (Stata Corp., College Station, United States).

Descriptive analyses were performed with absolute and relative frequency for categorical variables, and with mean for continuous variables. ANOVA or *t*-test were performed for comparison of means and Chi-square was run for categorical variables to identify potential differences between groups, according to social vulnerability indicators.

The sample size calculation was performed a posteriori, using the G*Power application. With an alpha of 0.05, sample size of 924 and using the t-test and ANOVA for group comparison, this power analysis revealed a value of 0.99, which is adequate to conduct the analyses presented in the study (29).

The study was approved by the Research Ethics Committee of the Pedro Ernesto University Hospital (HUPE/UERJ) (CAAE: 54239621.4.0000.5259). The Informed Consent to participate was obtained from all participants prior to the study. The declaration was presented at the beginning of the online questionnaire; the participants could only answer the survey if the clicked on “I accept to participate.” This way, they were aware that all data would be used for research purposes only.

## 3 Results

Among a total of 4,751 students beginning their undergraduate studies in 2022, 1,111 (23.4%) agreed to participate, and 924 (83.2%) students were eligible. From those 75% of them studied on the Maracanã campus and 43% were from the area of Social Sciences, which covers the courses of business management, archeology, accounting sciences, economic sciences, social sciences, law, philosophy, history, international relations, and social service. Most of them were 18–22 years-old (50%), cisgender women (62%), white people (53%), 30% of these students had been admitted through the quota system, 38% said that their mothers’ level of education was complete or incomplete higher education. 88% lived with relatives that were not necessarily their own parents, and 32% indicated that family income ranged from 2 to 5 minimum wages (Table 1).

**Table 1:** Characterization of students admitted in a Brazilian public university in 2022, Rio de Janeiro, 2022.

Characteristics	*N*	%
Campus		
Maracanã	697	75.4
Other campuses	227	24.6
Undergraduate course area		
Biomedical	137	14.9
Education and humanities	104	11.3
Social sciences	394	42.8
Technology and science	286	31.1
Age		
18–22 years	453	50.1
23–29 years	234	25.9
30–71 years	217	24.0
Gender		
Cisgender woman	576	62.3
Cisgender man	295	31.9
Transgender man	3	0.3
Non-binary person	22	2.4
Other	13	1.4
Rather not say	15	1.6
Race/skin color		
White	485	52.5
Brown	235	25.4
Black	194	21.0
Asian	10	1.1
Paid activity		
Yes	332	35.9
Household arrangement		
Living alone	90	9.8
Living with relatives, not necessarily parents	811	88.1
Living with other people who are not family members	20	2.2
Mother’s level of education		
None	17	1.8
Incomplete elementary school	158	17.1
Complete elementary school/incomplete high school	109	11.8
Complete high school	280	30.3
Complete or incomplete higher education	352	38.1
Unsure	8	0.9
Family income		
Up to 1 minimum wage	103	11.2
From 1–2 minimum wages	181	19.6
From 2–5 minimum wages	295	31.9
From 5–10 minimum wages	154	16.7
More than 10 minimum wages	191	20.7
Quota		
Yes	278	30.1
Socioeconomic score		
1st tertile	277	39.9
2nd tertile	187	27.0
3rd tertile	230	33.1
Food and nutrition security situation		
Food secure	591	64.0
Food insecurity without hunger	196	21.2
Food insecurity with hunger	137	14.8

The food and nutrition security situation of university students was associated to the socioeconomic score and form of university admission. More than 50% of students in the first tertile of the score (i.e., at the lowest level) were food insecure (with or without hunger). The frequency of food insecurity decreased with an increased socioeconomic score, namely 56% food insecurity in the first tertile (with or without hunger) and 11% in the third tertile. Considering the form of admission, more than half of the quota-system students were food insecure (with or without hunger), compared to 28.8% of non-quota students (Table 2).

**Table 2:** Food and nutrition security situation of university students according to socioeconomic score and form of admission, Rio de Janeiro, 2022.

Food and nutrition security situation	Socioeconomic score %				Form of university admission %		
	T1 (*n* = 277)	T2 (*n* = 187)	T3 (*n* = 230)	*p*-value*	Non-quota (*n* = 646)	Quota (*n* = 278)	*p*-value*
Food secure	44.4	61.5	89.1	<0.001	71.4	46.8	<0.001
Food insecurity without hunger	27.8	26.7	7.4		16.9	31.3	
Food insecurity with hunger	27.8	11.8	3.5		11.8	21.9	

T, tertile.

* *p*-value according to chi-square.

The mean score for consumption of fresh or minimally processed food decreased with the decrease of the socioeconomic score, from 7.2 (T3) to 6.5 (T1) (*p* = 0.002), but did not differ according to the form of admission. Students in food-secure or food-insecure without hunger presented a higher consumption (6.9), compared to those food-insecure with hunger (6.0) (*p* = 0.002). The ultra-processed food score did not differ according to socioeconomic score, form of admission to university or food security situation (Table 3).

**Table 3:** Mean scores of food consumption of university students by socioeconomic score, form of admission and food and nutritional security situation, Rio de Janeiro, 2022.

Consumption on the previous day	Socioeconomic score				Form of university admission			Food and nutritional security situation			
	T1 (*n* = 277)	T2 (*n* = 187)	T3 (*n* = 230)	*p*-value*	Non-quota (*n* = 646)	Quota (*n* = 278)	*p*-value**	Food secure (*n* = 591)	Food insecurity without hunger (*n* = 196)	Food insecurity with hunger (*n* = 137)	*p*-value*
Fresh or minimally processed foods											
Mean score	6.5	6.7	7.2	0.002	6.7	6.8	0.733	6.9	6.7	6.0	0.002
Ultra-processed foods											
Mean score	4.5	4.8	4.5	0.830	4.4	4.6	0.290	4.5	4.8	4.1	0.160

T, tertile.

* *p*-value according to ANOVA.

** *p*-value according to t test.

Considering the regular intake of fresh or minimally processed foods, for vegetables (T1: 35% vs. T3: 51%) (*p* = 0.001) and fruits (T1: 18% vs. T3: 33%) (*p* < 0.001), there was a higher frequency of intake in the third tertile of the socioeconomic score when compared to the first tertile. The frequency of regular intake of vegetables among non-quota students (45%) was also higher when compared to quota ones (35%) (*p* = 0.003). The analysis of the relationship between food consumption and food and nutritional security showed noteworthy results for fresh or minimally processed foods. Regular intake of vegetables and fruits was more frequent among food-secure (48 and 31%, respectively), when compared to food-insecure students without hunger (36 and 17%, respectively) or with hunger (27 and 16%, respectively) (*p* < 0.001). Fruit juice was more often consumed by food-secure students (10%) than food-insecure ones with hunger (1%) (*p* < 0.001) (Table 4).

**Table 4:** Frequency of regular food consumption of university students according to socioeconomic score, form of admission and food and nutritional security situation, Rio de Janeiro, 2022.

Regular consumption	Frequency (%)										
	Socioeconomic score				Form of university admission			Food and nutritional security situation			
	T1 (*n* = 277)	T2 (*n* = 187)	T3 (*n* = 230)	*p*-value*	Non-quota (*n* = 646)	Quota (*n* = 278)	*p*-value*	Food secure (*n* = 591)	Food insecurity without hunger (*n* = 196)	Food insecurity with hunger (*n* = 137)	*p*-value*
Fresh or minimally processed foods											
Bean	59.2	57.8	55.2	0.662	54.3	61.2	0.055	55.7	62.8	50.4	0.068
Vegetables	35.4	42.2	51.3	0.001	45.4	34.9	0.003	47.9	35.7	27.0	<0.001
Fruit	17.7	29.9	33.0	<0.001	27.6	21.9	0.074	31.1	16.8	16.1	<0.001
Natural fruit juice	5.8	9.1	9.6	0.229	8.8	5.4	0.075	10.0	6.1	0.7	0.001
Ultra-processed foods											
Fried snack	3.6	0.5	1.3	0.044	2.3	2.5	0.858	2.4	1.5	3.6	0.459
Processed meats	4.7	7.0	3.5	0.258	5.6	5.0	0.741	5.2	8.2	2.2	0.058
Packed salted	6.5	9.6	9.6	0.353	7.9	7.6	0.859	8.5	7.1	5.8	0.546
Sweet cookie	5.8	7.0	6.1	0.873	6.0	5.8	0.868	5.2	7.7	6.6	0.442
Salty biscuit	6.1	4.8	6.5	0.746	5.1	5.8	0.687	4.9	7.1	4.4	0.419
Sweets	13.0	16.6	20.9	0.060	18.3	14.7	0.194	19.8	15.3	8.8	0.006
Canned, bottled or powdered juice	7.6	4.3	8.3	0.240	5.7	11.1	0.004	7.4	10.2	2.9	0.043
Soft drink	9.4	9.6	11.3	0.752	9.9	9.0	0.666	9.5	9.2	10.9	0.846

T, tertile.

* *p*-value according to chi-square.

Regarding the intake of ultra-processed foods, fried snacks were consumed more by the first third (T1 3.6 vs. T2 0.5 vs. T3 1.3; *p* = 0.044) and only canned/bottled/powdered juice was most frequently consumed by quota students (11%) than by non-quota ones (6%) (*p* = 0.004). Sweets were more often consumed by food-secure (20%) than by food-insecure students with hunger (9%) (*p* = 0.006) and canned/bottled/powdered juice was more frequently consumed by those in a food insecure situation without hunger (10%) than by food secure students (7%) and in food insecure with hunger (3%) (*p* = 0.043). Contrasting the frequency of report of ultra-processed items between the food-insecure students without and those with hunger, the former showed higher frequency of intake of sweets, canned/bottled/powdered juice, salty biscuit, and processed meats - items that are typically eaten for a snack. The food-insecure students with hunger reported the consumption of fried snack foods more often, likely as a meal substitute (Table 4).

## 4 Discussion

The evaluation of food consumption by students who entered university in 2022, after the COVID-19 pandemic, reveals that situations related to social vulnerability influenced their food consumption. Importantly, considering the composed indicators of vulnerability used, about a third of the students could be considered socially vulnerable. Fresh or minimally processed foods, in general, were more consumed by students with higher socioeconomic score, non-quota students and by food-secure ones. For ultra-processed foods, the differences seem to be less influenced by the socioeconomic situation in this sample of students. However, it is important to note that the lack of significant differences in the ultra-processed food consumption may also reflect limitations of the measurements adopted.

Little difference was found in the consumption of ultra-processed foods between the different forms of assessing vulnerability. This is probably due to the wide availability of these foods and the increase in food prices, one of the impacts of the COVID-19 pandemic (30). A study conducted in Australia found that the price of healthy foods increased by about 12.8% while that of unhealthy foods increased by 7.0% between 2021 and 2022 (31). In Brazil, this is likely to be applicable as well. Ultra-processed foods were more expensive (R$ 6.51/kg) in 1995 than fresh or minimally processed foods and culinary ingredients (R$ 3.45/kg); however, the price of ultra-processed foods has been decreased since the 2000s; they became cheaper, decreasing the price gap between them and fresh or minimally processed foods. Furthermore, ultra-processed foods were expected to become cheaper than fresh or minimally processed foods in 2026 (32). This trend may have been anticipated because of the pandemic. Also, there was an increase in the energy content of ultra-processed foods in household purchases between 2002 and 2018 (12.6% vs. 18.4%) and a decrease in the purchase of fresh or minimally processed foods (53.3% vs. 49.5%). Despite the lower share of ultra-processed foods among low-income households, there has been a more intense increase in the share of them among these households over the years (7, 33).

A literature review pointed out that most university students showed unhealthy eating practices, characterized by high consumption of ultra-processed foods and low consumption of fruits, vegetables, fish, whole grains and legumes (10). These data corroborate the findings of the present study. At least in part, such practices may be related to changes related to the entrance in the university, e.g., leaving their parents’ house, becoming responsible for their own food, performing financial management (34, 35). However, the present study analyzed students at their admission to university; thus, such changes may not have affected them yet. Low consumption of fresh or minimally processed foods is common among university students (9–11). There was a difference between the social groups studied for foods such as vegetables and fruits, despite their importance in people’s diet together with other fresh or minimally processed foods (36).

From the perspective of social vulnerability, in 2000, Act no. 3.524 established a quota system in state universities of Rio de Janeiro, which was updated in 2018 by Act no. 8.121 (37). In 2003, UERJ, the scenario of the present study, implemented the quota system for admission via college entrance exam, based on different criteria: economic (income), racial (black and brown), education (students from publicly-funded schools), among others (27). The quota policy is a social inclusion policy that aims to promote equality, reducing social injustices and ensuring access to and permanence at university (38). However, these students must be ensured adequate conditions to remain at university, including the guarantee of adequate and healthy eating.

The university quota-system in Brazil, in addition to income, can also consider race, which may reflect an even greater social vulnerability, especially of black people owing to the slave history and its consequences in Brazilian society. Among the 10% poorest people in Brazil, blacks tend to be poorer than whites; therefore, they are more subject to food insecurity (39). A Brazilian data from 2017 to 2018 showed that food insecurity was present in 45.6% of households where black men or women were the heads, while this frequency was 26.1% in households headed by white people (33). A study with Brazilian university students showed that socioeconomic status and race/color seem to determine a lower frequency of consumption of healthy food markers, e.g., vegetables (37). Although we have opted for composed indicators of social vulnerability, and did not explore the race/color variable directly but only as part of the quota variable, it is essential to recognize the potentially greater social vulnerability of the quota students, not only because of their lower income, but also for the structural racism existing in the country.

Even though the quotas were a marker of vulnerability, there was not a great difference between the consumption of fresh or minimally processed foods of quota and non-quota students. This result corroborates the findings of the study of Perez et al., in which quota students and non-quota students had similar eating practices (8). Nevertheless, more than half of the quota students were food insecure, which is another marker of vulnerability. A US study that evaluated university students found that 15% of them were food insecure and 16% were at risk of food insecurity, which amounts to less than the rates found in the present study. However, they also found that African-American students of another race/ethnicity, who received financial aid or had housing problems, were more likely to experience food insecurity or risk of food insecurity, which coincides with the proposition of Brazilian quotas, that is, non-white students with worse socioeconomic conditions would be more likely to be food insecure (40).

Food insecurity among university students is an important social problem, which may have implications for academic performance and student permanence in the institution until completion of the degree course. Thus, awareness of food and nutritional security at universities may strengthen accessibility policies and student permanence. The data from the present study on frequency of food insecurity (36%) are similar to those of the national data of 2017–2018 (36.7%), of university students in Canada (37.2%) and Australian university students (41.9%) (33, 41, 42). One study found that food-insecure students from a US university were more likely to be among the 10% lowest yield coefficients (YC) and less likely to be among the highest 10% YC (43).

The consumption of fresh or minimally processed foods was significantly higher among food-secure students, which makes sense, since food insecurity involves quantitative and qualitative food restriction. A systematic review that evaluated the association between food insecurity and food outcomes among university students found convergent data: food-insecure students presented lower consumption of healthy foods (such as fruits, vegetables, and whole grains) and higher consumption of unhealthy foods (e.g., fast foods). Despite the lack of difference in the score for intake of ultra-processed foods, it is worth mentioning that food-insecure students had a higher frequency of intake of some ultra-processed items, but with differences between those with hunger, with a profile of ultra-processed foods for meal replacement, and those without hunger, with a profile for intake of snacks. In this sense, the most vulnerable groups may be “protected” from the ultra-processed ones because they need to prioritize meals rather than snacks.

The finding that ultra-processed food consumption was less influenced by social vulnerability can be considered intriguing. It is necessary to mention that even if the consumption has been similar across groups, the reasons behind it might differ. For vulnerable students, the consumption of ultra-processed foods may reflect the high availability and affordability in their neighborhoods, together with a lack of access to fresh or minimally processed foods, and also their convenience, and lack of access to cooking facilities, whereas for more privileged students, it might be a choice.

Socioeconomic factors play a fundamental role in the physical, psychological and social development of adolescents, and socioeconomic inequalities are important social determinants of the health of the general population and of this segment in particular (44–47). This scenario reinforces the importance of the data of the present study to guide actions and interventions geared for student permanence at universities and the promotion of a food environment that favors adequate and healthy eating. In this context, a central action can be the presence of a university restaurant on all campuses to be able to serve all students at the university, with subsidized or free food for vulnerable students.

The present study presents some limitations. The main one is the fact that the evaluated population is not representative of the students admitted in 2022, because although they were all contacted to participate in the study, about 20% answered the questionnaire. The response rate, although similar to other online surveys, also raises the possibility of selection bias, as students most affected by food insecurity might have been less likely to participate, potentially reinforcing this underestimation. However, it is worth mentioning that the distribution of our sample is similar to that observed among the enrolled students, even considering the quota students (14). The socioeconomic score questions were not mandatory, since the respondents in the pre-test reported discomfort in answering some of the questions. Thus, the score was calculated for 75% of the sample. It is possible that the vulnerability scenario and its consequences are even more intense than that shown in the study, due to the possible non-response bias of the socioeconomic score. The study was restricted to one Brazilian public university, but it is possible to infer that after the implementation of quota system also in federal university the sociodemographic characteristics of the students can be similar. Furthermore, other potential sources of bias should be acknowledged, such as the risk of social desirability due to self-reported data collected through an online questionnaire, the lack of adjustment for potential confounding factors, and the limitation of applying a food security scale designed for adolescents to a population of young adults. Finally, the cross-sectional design prevents establishing causal relationships, so the observed findings should be interpreted as associations.

Despite the limitations, the present study evaluated a relatively large sample of university students, compared to other studies with this population (48–51). The study focused on ultra-processed foods, but also in fresh or minimally processed foods and used two strategies to assess the consumption, on the previous day and the regular consumption. As mentioned before, both strategies are currently adopted in national health surveys (22, 23) and, additionally, the use of similar screeners was recommended by the World Health Organization to monitor healthy diets globally (52). The use of three composed indicators for the assessment of the social vulnerability soon after the return to classes and face-to-face activities, after the COVID-19 pandemic, also should be highlighted, since it can be useful to identify more vulnerable groups and support the design of interventions for them in the context of the university. The scale used to measure food and nutrition security is validated and proposes three levels of classification of individuals, including the categories food insecurity without hunger and food insecurity with hunger. However, it should be considered that the fact of being afraid of hunger already represents a situation of vulnerability and violation of the human right to adequate and healthy eating.

In conclusion, the consumption of fresh or minimally processed foods varied according to the situation of social vulnerability of university students. The data reinforce how much student permanence policies are needed to support students. This is a population which spends part of the day or the whole day at university, so these sites can be strategic for promoting food and nutrition security and for ensuring the human right to adequate and healthy food within the university food environment.

## Data Availability

The raw data supporting the conclusions of this article will be made available by the authors, without undue reservation.
